# Impact of TOPAZ-1 eligibility on the survival benefit of durvalumab plus gemcitabine–cisplatin in advanced biliary tract cancer: a multicenter real-world study

**DOI:** 10.1007/s00535-026-02412-6

**Published:** 2026-04-12

**Authors:** Hisashi Kosaka, Rie Sugimoto, Masahiko Kinoshita, Satoshi Yasuda, Satoru Kakizaki, Jun Sakata, Kiyonori Yamai, Takeshi Hatanaka, Yusuke Yamamoto, Toshifumi Sato, Toru Ishikawa, Takanori Morikawa, Jun Hanaoka, Haruki Mori, Hidenori Toyoda, Tsuyoshi Sanuki, Misaki Yokoi, Hiroyuki Shibata, Koji Fukuda, Kazuhito Kawata, Koji Amaya, Takashi Ito, Masaaki Hidaka, Atsushi Naganuma, Keishi Sugimachi, Masaki Kaibori

**Affiliations:** 1 Department of Hepatobiliary Surgery, Kansai Medical University, 2-5-1, Shin-machi, Hirakata, Osaka 573-1010 Japan; 2 Department of Hepato-Biliary-Pancreatology, NHO Kyushu Cancer Center, Fukuoka, Japan; 3 Department of Hepatobiliary–Pancreatic Surgery, Osaka Metropolitan University, Osaka, Japan; 4 Department of Surgery, Nara Medical University, Kashihara, Japan; 5 Department of Clinical Research, NHO Takasaki General Medical Center, Takasaki, Japan; 6 Division of Digestive and General Surgery, Niigata University Graduate School of Medical and Dental Sciences, Niigata, Japan; 7 Department of Gastroenterology, Hematology and Oncology, Odate Municipal General Hospital, Odate, Japan; 8 Department of Gastroenterology, Gunma Saiseikai Maebashi Hospital, Maebashi, Japan; 9 Department of Digestive Surgery, Kyoto Prefectural University of Medicine, Kyoto, Japan; 10 Department of Gastroenterology, Saiseikai Niigata Hospital, Niigata, Japan; 11 Department of Surgery, Miyagi Cancer Center, Natori, Japan; 12 Department of Gastroenterological Surgery, Ehime Prefectural Central Hospital, Matsuyama, Japan; 13 Department of Surgery, Shiga University of Medical Science, Otsu, Japan; 14 Department of Gastroenterology and Hepatology, Ogaki Municipal Hospital, Ogaki, Japan; 15 Department of Gastroenterology, Hyogo Prefectural Harima-Himeji General Medical Center, Himeji, Japan; 16 Department of Clinical Oncology, Akita University Graduate School of Medicine, Akita, Japan; 17 Hepatology Division, Department of Internal Medicine II, Hamamatsu University School of Medicine, Hamamatsu, Japan; 18 Department of Surgery, Toyama Prefectural Central Hospital, Toyama, Japan; 19 Third Department of Internal Medicine, Division of Gastroenterology and Hepatology, Kansai Medical University, 2-5-1, Shin-machi, Hirakata, Osaka 573-1010 Japan; 20 Digestive and General Surgery, Faculty of Medicine, Shimane University, Izumo, Japan; 21 Department of Gastroenterology, NHO Takasaki General Medical Center, Takasaki, Japan; 22 Department of Hepatobiliary and Pancreatic Surgery, NHO Kyushu Cancer Center, Fukuoka, Japan

**Keywords:** Biliary tract neoplasms, Durvalumab, Gemcitabine, Cisplatin, Survival analysis

## Abstract

**Background:**

Durvalumab plus gemcitabine–cisplatin (GCD) has become a standard first-line therapy for advanced biliary tract cancer (BTC) following the TOPAZ-1 trial. However, whether the survival benefit observed in trial-eligible patients can be generalized to broader real-world populations remains uncertain. We evaluated the impact of TOPAZ-1 eligibility on the effectiveness of GCD in routine clinical practice.

**Methods:**

In this multicenter retrospective cohort study, 610 patients with unresectable or recurrent BTC treated with first-line GCD (*n* = 268) or gemcitabine–cisplatin (GC) (*n* = 342) at 19 Japanese institutions were analyzed. Patients were classified according to TOPAZ-1 eligibility criteria. Overall survival (OS) was compared between treatment groups in the entire cohort and stratified by eligibility status. Multivariable Cox models were constructed separately for eligible and ineligible patients.

**Results:**

Among 610 patients, 324 (53.1%) met TOPAZ-1 eligibility criteria. In the overall cohort, GCD was associated with longer OS than GC (median, 13.7 vs 11.3 months; *p* = 0.009). Among eligible patients, GCD significantly improved OS compared with GC (18.0 vs 13.1 months; *p* = 0.004), whereas no significant difference was observed among ineligible patients (10.8 vs 10.0 months; *p* = 0.675). However, the interaction between treatment and TOPAZ-1 eligibility was not statistically significant (*p* for interaction = 0.162).

**Conclusions:**

In this real-world cohort, the survival benefit of GCD appeared to be primarily observed in patients meeting TOPAZ-1 eligibility criteria. Trial-based eligibility may influence the magnitude of benefit from immunochemotherapy in advanced BTC, underscoring the importance of patient selection in routine practice.

**Supplementary Information:**

The online version contains supplementary material available at 10.1007/s00535-026-02412-6.

## Introduction

Biliary tract cancer (BTC) is an aggressive malignancy with a poor prognosis, as most patients are diagnosed at an advanced stage owing to nonspecific symptoms and anatomical complexity [1, 2]. For more than a decade, gemcitabine plus cisplatin (GC) has been the standard first-line chemotherapy for advanced BTC following the ABC-02 trial, which demonstrated a modest but significant survival benefit compared with gemcitabine alone [3]. More recently, the phase 3 TOPAZ-1 trial showed that adding the anti-PD-L1 antibody durvalumab to GC (GCD) significantly improved overall survival, establishing GCD as a new standard of care [4].

However, patients enrolled in randomized clinical trials are highly selected according to predefined eligibility criteria, including performance status and organ function. Whether the survival benefit observed in TOPAZ-1 can be generalized to the broader population encountered in routine clinical practice remains uncertain. In real-world settings, patients with BTC are often older and present with biliary obstruction, impaired liver function, systemic inflammation, or high tumor burden, all of which may influence treatment tolerance and prognosis. Although several real-world studies have reported outcomes of GCD [5–8], most were single-arm analyses without a contemporaneous GC control group. A limited number of retrospective comparative studies have evaluated GCD versus GC; however, baseline imbalances and temporal bias were not fully addressed [9]. Importantly, no prior studies have examined whether the effectiveness of GCD differs according to TOPAZ-1 eligibility status in real-world practice.

Accordingly, this multicenter study evaluated overall survival and safety outcomes between GCD and GC as first-line therapy for unresectable or recurrent BTC. Patients were stratified by TOPAZ-1 eligibility to determine whether the survival benefit of GCD was confined to trial-eligible patients or extended to those who would have been ineligible for the pivotal trial.

## Materials and methods

### Study design and patients

**Fig. 1:**
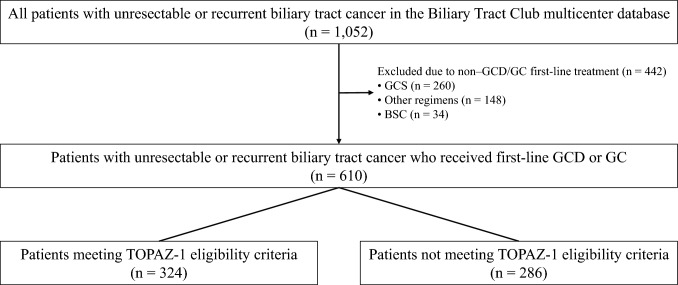
Flow diagram of patient selection. Flow diagram of patient selection from the Biliary Tract Club multicenter database. Among 1,052 patients with unresectable or recurrent biliary tract cancer, 442 patients who did not receive first-line GC or GCD were excluded. The remaining 610 patients were classified according to TOPAZ-1 eligibility status. *GC* gemcitabine plus cisplatin, *GCD* durvalumab plus gemcitabine and cisplatin, *GCS* gemcitabine plus cisplatin plus S-1, *BSC* best supportive care, *TOPAZ-1* a phase 3 trial of durvalumab plus gemcitabine and cisplatin in advanced biliary tract cancer

This multicenter retrospective cohort study was conducted using the Biliary Tract Club database, including patients with unresectable or recurrent BTC treated at 19 institutions in Japan between 2017 and 2024. Among 1,052 patients who received systemic therapy during the study period, 610 patients treated with first-line GCD or GC were included in the present analysis. Patients receiving other first-line regimens were excluded (Fig. 1). The included cohort comprised 268 patients treated with GCD and 342 treated with GC. Patients were further classified according to TOPAZ-1 eligibility criteria to evaluate the generalizability of trial-based evidence to routine clinical practice. Baseline demographic, clinical, and laboratory data were retrospectively collected from electronic medical records. The study was approved by the Institutional Review Board of Kansai Medical University (Approval No. 2023320) and conducted in accordance with the Declaration of Helsinki.

### Definitions of unresectable biliary tract cancer

Unresectability was determined at each institution by a multidisciplinary team based on radiologic and clinical findings. Disease was considered unresectable according to technical, functional, or oncological factors. Technical factors included unreconstructable biliary involvement or major vascular invasion precluding R0 resection. Functional factors included inadequate future liver remnant, impaired hepatic reserve (ICG-Krem < 0.05–0.06), or poor performance status. Oncological factors included distant metastasis or extensive nodal disease considered unsuitable for curative-intent surgery.

### Chemotherapy regimens

The GC regimen consisted of gemcitabine (1000 mg/m^2^) and cisplatin (25 mg/m^2^) administered intravenously on days 1 and 8 of a 21-day cycle. The GCD regimen consisted of durvalumab (1500 mg) administered intravenously every 3 weeks in combination with gemcitabine and cisplatin at the same doses and schedule as GC [4]. Dose reductions, treatment delays, and modifications were performed at the discretion of the treating physicians according to institutional standards.

### Outcome measures

The primary endpoint was overall survival (OS), defined as the time from initiation of first-line chemotherapy to death from any cause or last follow-up. Median follow-up time was estimated using the reverse Kaplan–Meier method. Progression-free survival (PFS) was defined as the time from treatment initiation to radiologic disease progression or death from any cause. Tumor response was evaluated according to the Response Evaluation Criteria in Solid Tumors (RECIST) version 1.1 in patients with assessable imaging data. Adverse events were graded according to the Common Terminology Criteria for Adverse Events (CTCAE) version 5.0. Adverse event analyses focused on clinically relevant toxicities, and data on Grade ≥ 3 events were systematically collected; lower-grade (Grade 1–2) adverse events were not consistently documented and were, therefore, not included in the analysis. Relative dose intensity (RDI) was calculated across all regimen components over the treatment period. The regimen included gemcitabine, cisplatin, and durvalumab, each contributing to the total dose intensity based on their standard dosing patterns. The dose intensity of each agent was defined according to its standard dosing, and the total dose intensity was determined by summing across all components over the actual treatment duration. The overall RDI was then calculated as the ratio of the total delivered dose intensity to the total planned dose intensity. After completion of the combination phase, subsequent treatment reflected routine clinical practice and varied across institutions in this retrospective cohort. Some patients continued durvalumab monotherapy as maintenance in accordance with the TOPAZ-1 protocol, whereas others switched to alternative regimens according to clinical judgment.

### TOPAZ-1 eligibility criteria

TOPAZ-1 eligibility was defined according to the original trial criteria [4], including age ≥ 18 years, ECOG performance status 0–1, measurable disease per RECIST 1.1, no prior immune checkpoint inhibitor exposure, exclusion of ampullary carcinoma, and adequate organ function. Adequate organ function was defined as hemoglobin ≥ 9.0 g/dL, absolute neutrophil count ≥ 1.5 × 10⁹/L, platelet count ≥ 100 × 10⁹/L, total bilirubin ≤ 2.5 × the upper limit of normal (ULN), AST and ALT ≤ 2.5 × ULN, and creatinine clearance > 50 mL/min. Patients not fulfilling any of these criteria were classified as TOPAZ-1 ineligible. Life expectancy < 12 weeks was retrospectively determined based on clinical documentation in the medical records, including physician assessment and relevant clinical factors at treatment initiation. Definitions were aligned with the published TOPAZ-1 protocol as closely as possible within the constraints of retrospective data.

### Statistical analysis

Categorical variables were compared using the *χ*^2^ test or Fisher’s exact test, as appropriate. Continuous variables were compared using the Mann–Whitney *U* test. Survival curves were estimated using the Kaplan–Meier method and compared using the log-rank test. OS was analyzed in the entire cohort and stratified according to TOPAZ-1 eligibility status. Prespecified subgroup analyses were performed according to TOPAZ-1 eligibility (eligible vs ineligible). To reduce the influence of early deaths and to confirm the robustness of the observed survival differences, a 6-month landmark analysis was conducted, including only patients alive at 6 months and evaluating subsequent survival from that time point. Multivariable Cox proportional hazards models were constructed separately for patients meeting and not meeting TOPAZ-1 eligibility criteria to identify independent prognostic factors and evaluate the treatment effect of GCD versus GC after adjustment for clinically relevant baseline covariates. Covariates included age, sex, primary tumor site, recurrent disease status, distant metastasis, biliary drainage, and log-transformed CA19-9. Hazard ratios (HRs) and 95% confidence intervals (CIs) were calculated. An interaction term between treatment and TOPAZ-1 eligibility was included in the Cox proportional hazards model to assess effect modification. An additional exploratory multivariable Cox analysis was performed in patients classified as TOPAZ-1 ineligible, including individual reasons for ineligibility and the presence of multiple eligibility violations (≥ 2 vs 1) as covariates. All statistical analyses were performed using IBM SPSS Statistics version 22.0 (IBM Japan Ltd., Tokyo). A two-sided *p* value < 0.05 was considered statistically significant.

## Results

### Patient characteristics

A total of 610 patients who received first-line GCD or GC were included in the analysis. Baseline characteristics are summarized in Table 1. Among them, 324 patients (53.1%) met the TOPAZ-1 eligibility criteria, whereas 286 (46.9%) were classified as ineligible. Compared with TOPAZ-1 eligible patients, those who were ineligible were significantly older (70.5 vs 73.0 years, *p* < 0.001) and had poorer baseline organ function. Hemoglobin and albumin levels were lower in the ineligible group, whereas total bilirubin and AST levels were higher, and creatinine clearance was lower. The ALBI score was also worse in the ineligible cohort (all *p* < 0.001). In addition, although all eligible patients had an ECOG performance status of 0–1 by definition, 12.2% of ineligible patients had a performance status ≥ 2 (*p* < 0.001). There were no significant differences in sex distribution, recurrent disease status, distant metastasis, platelet count, or C-reactive protein levels between the two groups. Ampullary carcinoma was observed exclusively in the ineligible cohort. The proportion of patients treated with GCD did not significantly differ between eligible and ineligible patients. The reasons for TOPAZ-1 ineligibility are summarized in Supplementary Table 1. The most common causes included impaired renal function (creatinine clearance ≤ 50 mL/min), elevated liver enzymes (AST or ALT > 2.5 × ULN), and non-measurable disease.

**Table 1:** Baseline characteristics of patients according to TOPAZ-1 eligibility status

Variable	Overall (*n* = 610)	TOPAZ-1 eligible (*n* = 324)	TOPAZ-1 ineligible (*n* = 286)	*p* value
Age	72.0 (66.0–76.0)	70.5 (64.0–75.0)	73.0 (68.0–78.0)	< 0.001
Sex, male	382 (62.6)	200 (61.7)	182 (63.6)	0.627
ECOG PS, 0–1	575 (94.3)	324 (100.0)	251 (87.8)	< 0.001
Primary tumor site				< 0.001
PHC	164 (26.9)	81 (25.0)	83 (29.0)	
ICC	174 (28.5)	106 (32.7)	68 (23.8)	
GBC	137 (22.5)	82 (25.3)	55 (19.2)	
DCC	100 (16.4)	55 (17.0)	45 (15.7)	
AC	35 (5.7)	0 (0.0)	35 (12.2)	
Recurrent tumor	214 (35.1)	114 (35.2)	100 (35.0)	0.955
Distant metastasis	437 (71.6)	242 (74.7)	195 (68.2)	0.075
Biliary drainage	236 (38.7)	111 (34.3)	125 (43.7)	0.017
Hemoglobin, g/dL	12.0 (10.7–13.2)	12.5 (11.1–13.5)	11.5 (10.2–12.7)	< 0.001
Platelet count, × 10^4^/μL	23.1 (17.2–29.7)	23.1 (17.5–29.1)	23.2 (16.9–30.6)	0.566
Total bilirubin, mg/dL	0.7 (0.5–1.1)	0.7 (0.5–0.9)	0.8 (0.5–1.3)	< 0.001
Albumin, g/dL	3.6 (3.2–4.0)	3.7 (3.3–4.0)	3.5 (3.1–3.9)	< 0.001
AST, U/L	30.0 (22.0–45.0)	28 (21.0–37.3)	34.0 (24.0–66.0)	< 0.001
CrCl, mL/min	68.6 (55.4–85.2)	73.7 (63.3–88.7)	60.0 (47.6–78.8)	< 0.001
CRP, mg/dL	0.48 (0.16–2.08)	0.46 (0.15–1.80)	0.56 (0.17–2.33)	0.301
ALBI score	− 2.36 (− 2.70 to − 1.91)	− 2.51 (− 2.74 to − 2.05)	− 2.19 (− 2.60 to − 1.79)	< 0.001
CA19-9, U/mL	152.2 (26.4–1235.0)	116.5 (21.6–1136.0)	177.0 (32.5–1504.5)	0.029
GCD treatment	268 (43.9)	151 (46.6)	117 (40.9)	0.157

Values are expressed as number (percentage) or median (interquartile range)

*p* values were calculated using the Chi-square test for categorical variables and the Mann–Whitney *U* test for continuous variables, as appropriate

*ECOG PS* Eastern Cooperative Oncology Group performance status, *PHC* perihilar cholangiocarcinoma, *ICC* intrahepatic cholangiocarcinoma, *GBC* gallbladder cancer, *DCC* distal cholangiocarcinoma, *AC* ampullary cancer, *AST* aspartate aminotransferase, *CrCl* creatinine clearance, *CRP* C-reactive protein, *ALBI* albumin–bilirubin, *CA19-9 *carbohydrate antigen 19–9, *GCD* gemcitabine plus cisplatin plus durvalumab, *GC* gemcitabine plus cisplatin

### Overall survival in the entire cohort

**Fig. 2:**
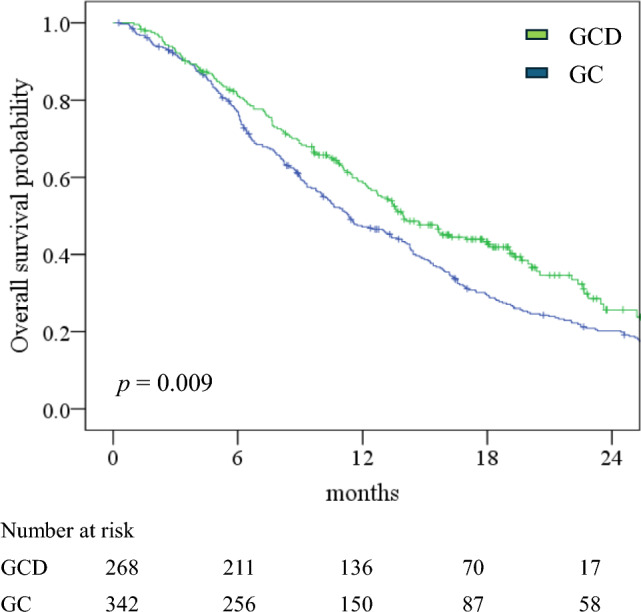
Overall survival in the entire cohort. Kaplan–Meier curves for overall survival comparing gemcitabine/cisplatin plus durvalumab (GCD) and gemcitabine/cisplatin (GC). *GCD* gemcitabine/cisplatin plus durvalumab, *GC* gemcitabine plus cisplatin

During a median follow-up of 44.3 months in the GC group and 19.2 months in the GCD group, treatment with GCD was associated with longer overall survival compared with GC (log-rank *p* = 0.009; Fig. 2). The median overall survival was 13.7 months (95% CI 11.8–15.5) in the GCD group and 11.3 months (95% CI 9.7–13.0) in the GC group. The survival curves separated early after treatment initiation and remained apart throughout follow-up.

### Overall survival and progression-free survival stratified by TOPAZ-1 eligibility

**Fig. 3:**
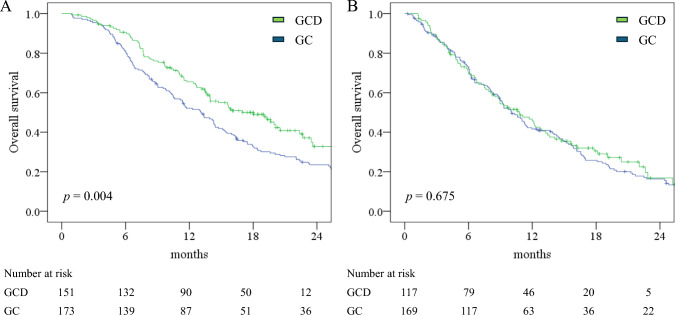
Overall survival according to treatment regimen stratified by TOPAZ-1 eligibility. Kaplan–Meier curves comparing overall survival between patients treated with gemcitabine plus cisplatin with durvalumab (GCD) and gemcitabine plus cisplatin (GC) among patients meeting TOPAZ-1 eligibility criteria (**A**) and those not meeting TOPAZ-1 eligibility criteria (**B**). *p* values were calculated using the log-rank test. *GC* gemcitabine plus cisplatin, *GCD* durvalumab plus gemcitabine and cisplatin, *TOPAZ-1* a phase 3 trial of durvalumab plus gemcitabine and cisplatin in advanced biliary tract cancer

When stratified by TOPAZ-1 eligibility status (Fig. 3), treatment with GCD was associated with longer OS compared with GC among patients meeting the TOPAZ-1 criteria, whereas no significant association was observed among ineligible patients. Among TOPAZ-1-eligible patients (Fig. 3A), median OS was 18.0 months (95% CI 13.8–22.2) in the GCD group and 13.1 months (95% CI 11.0–15.2) in the GC group (log-rank *p* = 0.004). In contrast, among TOPAZ-1-ineligible patients (Fig. 3B), median OS was 10.8 months (95% CI 8.3–13.2) in the GCD group and 10.0 months (95% CI 8.5–11.6) in the GC group (log-rank *p* = 0.675). Within each treatment group, TOPAZ-1-eligible status was associated with longer OS compared with ineligible status (Supplementary Figure 1).

PFS showed a broadly consistent direction of effect (Supplementary Figure 2). Among TOPAZ-1-eligible patients, median PFS was 7.9 months (95% CI 6.3–9.6) in the GCD group and 5.4 months (95% CI 4.4–6.4) in the GC group, although this difference did not reach statistical significance (log-rank *p* = 0.069). In contrast, no difference in PFS was observed among TOPAZ-1-ineligible patients (median, 5.6 vs 5.3 months; log-rank *p* = 0.933).

### Tumor response according to treatment regimen stratified by TOPAZ-1 eligibility

Tumor response was evaluable in 542 patients (Table 2). Among TOPAZ-1-eligible patients, objective response rates (ORR) were similar between the GCD and GC groups (15.9% vs 17.5%, *p* = 0.693), and disease control rates (DCR) were also comparable (74.2% vs 71.3%, *p* = 0.570). In the ineligible cohort, ORR remained lower overall and did not significantly differ between regimens (7.4% vs 11.2%, *p* = 0.366), with similar DCR (64.2% vs 64.8%, *p* = 0.928). Within the GCD-treated population, ORR was numerically higher in eligible than ineligible patients (15.9% vs 7.4%, *p* = 0.050).

**Table 2:** Tumor response according to RECIST version 1.1 in patients treated with gemcitabine–cisplatin plus durvalumab or gemcitabine–cisplatin, stratified by TOPAZ-1 eligibility

TOPAZ-1-eligible patients			
Variable	GCD (*n* = 151)	GC (*n* = 171)	*p* value
RECIST			0.690
CR	2 (1.3)	5 (2.9)	
PR	22 (14.6)	25 (14.6)	
SD	88 (58.3)	92 (53.8)	
PD	39 (25.8)	49 (28.7)	
ORR	24 (15.9)	30 (17.5)	0.693
DCR	112 (74.2)	122 (71.3)	0.570

TOPAZ-1-ineligible patients			
Variable	GCD (*n* = 95)	GC (*n* = 125)	*p* value
RECIST			0.417
CR	1 (1.1)	0 (0.0)	
PR	6 (6.3)	14 (11.2)	
SD	54 (56.8)	67 (53.6)	
PD	34 (35.8)	44 (35.2)	
ORR	7 (7.4)	14 (11.2)	0.366
DCR	61 (64.2)	81 (64.8)	0.928

RECIST-based analyses were performed in 542 patients with assessable radiologic response

Values are presented as number (percentage)

*p* values were calculated using the Chi-square test or Fisher’s exact test, as appropriate

*RECIST* Response Evaluation Criteria in Solid Tumors, *TOPAZ-1* a phase 3 trial of durvalumab plus gemcitabine and cisplatin in advanced biliary tract cancer, *GCD* gemcitabine plus cisplatin plus durvalumab, *GC* gemcitabine plus cisplatin, *CR* complete response, *PR* partial response, *SD* stable disease, *PD* progressive disease, *ORR* objective response rate, *DCR* disease control rate

### Safety and relative dose intensity stratified by TOPAZ-1 eligibility

Grade ≥ 3 neutropenia was more frequent with GCD than with GC in both TOPAZ-1-eligible (39.1% vs 22.0%, *p* = 0.001) and ineligible patients (29.1% vs 18.9%, *p* = 0.046). Grade ≥ 3 immune-related adverse events occurred only in the GCD group in both strata (eligible: 2.6% vs 0.0%, *p* = 0.031; ineligible: 3.4% vs 0.0%, *p* = 0.015). No significant differences were observed in grade ≥ 3 anemia or thrombocytopenia. Median RDI was comparable between regimens regardless of eligibility status (Table 3).

**Table 3:** Incidence of grade ≥ 3 adverse events and relative dose intensity in patients treated with gemcitabine–cisplatin plus durvalumab or gemcitabine–cisplatin, stratified by TOPAZ-1 eligibility

TOPAZ-1-eligible patients			
Variable	GCD (*n* = 151)	GC (*n* = 173)	*p* value
Neutropenia, grade ≥ 3	59 (39.1)	38 (22.0)	0.001
Anemia, grade ≥ 3	13 (8.6)	11 (6.4)	0.440
Thrombocytopenia, grade ≥ 3	9 (6.0)	7 (4.0)	0.428
Any irAE, grade ≥ 3	4 (2.6)	0 (0.0)	0.031
Relative dose intensity, %	84.5 (72.6 – 98.9)	82.0 (75.0 – 100.0)	0.810

TOPAZ-1-ineligible patients			
Variable	GCD (*n* = 117)	GC (*n* = 169)	*p* value
Neutropenia, grade ≥ 3	34 (29.1)	32 (18.9)	0.046
Anemia, grade ≥ 3	11 (9.4)	20 (11.8)	0.515
Thrombocytopenia, grade ≥ 3	11 (9.4)	10 (5.9)	0.267
Any irAE, grade ≥ 3	4 (3.4)	0 (0.0)	0.015
Relative dose intensity, %	80.0 (70.0 – 98.9)	82.3 (70.0 – 100.0)	0.345

Adverse events were graded according to the Common Terminology Criteria for Adverse Events

Relative dose intensity is presented as the median (interquartile range)

*p* values were calculated using the Chi-square test or Fisher’s exact test for categorical variables and the Mann–Whitney *U* test for continuous variables, as appropriate

*TOPAZ-1* a phase 3 trial of durvalumab plus gemcitabine and cisplatin in advanced biliary tract cancer, *GCD* gemcitabine plus cisplatin plus durvalumab, *GC* gemcitabine plus cisplatin, *irAE* immune-related adverse event

### Multivariable Cox analysis stratified by TOPAZ-1 eligibility

Multivariable Cox proportional hazards analyses stratified by TOPAZ-1 eligibility are shown in Table 4. Among patients meeting the TOPAZ-1 criteria, treatment with GCD was independently associated with improved OS compared with GC (HR, 0.709; 95% CI 0.532–0.946; *p* = 0.019). In contrast, among TOPAZ-1-ineligible patients, GCD was not significantly associated with OS (HR, 0.947; 95% CI 0.709–1.265; *p* = 0.713). In the eligible cohort, gallbladder cancer, recurrent disease status, and log-transformed CA19-9 were independently associated with survival. In the ineligible cohort, log CA19-9 remained a strong prognostic factor. The interaction between treatment and TOPAZ-1 eligibility was not statistically significant (*p* for interaction = 0.162). In an exploratory multivariable Cox analysis limited to TOPAZ-1-ineligible patients, hemoglobin < 9 g/dL (HR, 2.143; *p* = 0.021) and life expectancy < 12 weeks (HR, 6.462; *p* = 0.002) were independently associated with worse overall survival, whereas the presence of multiple eligibility violations (≥ 2 vs 1) showed only a trend toward worse survival (HR, 1.294; *p* = 0.074) (Supplementary Table 2).

**Table 4:** Multivariable Cox proportional hazards analyses for overall survival stratified by TOPAZ-1 eligibility status

Variable	TOPAZ-1-eligible HR (95% CI)	*p* value	TOPAZ-1-ineligible HR (95% CI)	*p* value
Age	1.013 (0.997–1.029)	0.123	0.998 (0.982–1.014)	0.798
Sex, male	1.141 (0.864–1.505)	0.353	1.172 (0.876–1.567)	0.286
Primary tumor site				
PHC	–	–	–	–
ICC	1.062 (0.716–1.574)	0.766	1.404 (0.925–2.131)	0.111
GBC	1.495 (1.007–2.220)	0.046	1.277 (0.826–1.975)	0.272
DCC	1.117 (0.709–1.758)	0.634	0.894 (0.564–1.415)	0.631
AC	–	–	1.102 (0.666–1.824)	0.705
Recurrent tumor	0.709 (0.519–0.970)	0.032	0.902 (0.635–1.282)	0.566
Distant metastasis	1.385 (0.991–1.937)	0.057	1.177 (0.863–1.604)	0.303
Biliary drainage	0.824 (0.592–1.147)	0.251	0.817 (0.577–1.158)	0.256
CA19-9 (log)	1.191 (1.064–1.333)	0.002	1.410 (1.232–1.614)	< 0.001
GCD treatment	0.709 (0.532–0.946)	0.019	0.947 (0.709–1.265)	0.713

Hazard ratios (HRs) and 95% confidence intervals (CIs) were estimated using multivariable Cox proportional hazards models, separately constructed for patients meeting and not meeting the TOPAZ-1 eligibility criteria

Perihilar cholangiocarcinoma was used as the reference category for primary tumor site

CA19-9 was analyzed after logarithmic transformation

*HR* hazard ratio, *CI* confidence interval, *PHC* perihilar cholangiocarcinoma, *ICC* intrahepatic cholangiocarcinoma, *GBC* gallbladder cancer, *DCC* distal cholangiocarcinoma, *AC* ampullary cancer, *CA19-9* carbohydrate antigen 19–9, *GCD* gemcitabine plus cisplatin plus durvalumab, *GC* gemcitabine plus cisplatin

### Six-month landmark analysis of overall survival

A 6-month landmark analysis was performed (Supplementary Figure S3). Among patients meeting the TOPAZ-1 eligibility criteria, treatment with GCD remained associated with longer overall survival compared with GC (log-rank *p* = 0.047). Median overall survival from the 6-month landmark was 13.6 months (95% CI 9.4–17.8) in the GCD group and 9.4 months (95% CI 7.6–11.2) in the GC group. In contrast, among TOPAZ-1-ineligible patients, no significant difference was observed between treatment groups (log-rank *p* = 0.406), with median overall survival of 9.6 months (95% CI 5.6–13.7) in the GCD group and 8.8 months (95% CI 6.2–11.5) in the GC group.

## Discussion

In this multicenter real-world cohort study including 610 patients with unresectable or recurrent BTC, treatment with GCD was associated with longer OS compared with GC in the overall population. Importantly, when stratified according to TOPAZ-1 eligibility status, this survival benefit was primarily observed among patients meeting the original trial criteria, whereas no significant survival difference was detected among those who would have been ineligible for the pivotal study. Although the survival benefit of GCD appeared to be more pronounced in TOPAZ-1-eligible patients, the interaction test did not reach statistical significance (*p* for interaction = 0.162). Therefore, a definitive difference in treatment effect between eligible and ineligible patients could not be confirmed, and these findings should be interpreted with caution. These exploratory findings further support the clinical heterogeneity of the TOPAZ-1-ineligible population. In particular, severe anemia and short estimated life expectancy were independently associated with worse survival, whereas the presence of multiple eligibility violations showed only a limited association with prognosis. This suggests that the impact of TOPAZ-1 ineligibility may depend more on the nature of the underlying clinical condition than simply the number of violated criteria.

The TOPAZ-1 trial established GCD as a new standard of care by demonstrating a sustained overall survival benefit with durable separation of survival curves over extended follow-up [4, 10]. However, randomized trials enroll highly selected patients with preserved performance status and organ function. Real-world BTC populations are frequently older and present with biliary obstruction, hepatic dysfunction, systemic inflammation, or comorbidities that would preclude trial participation. Several observational studies have reported outcomes of GCD in routine practice [5–9], yet most have not specifically examined whether trial eligibility modifies treatment effect. Our findings suggest that biological and functional characteristics linked to trial eligibility may modify the magnitude of benefit derived from immunochemotherapy.

In our cohort, TOPAZ-1-ineligible patients were older and had impaired organ function, reflected by lower hemoglobin and albumin levels, higher bilirubin and AST levels, and reduced creatinine clearance. Beyond their established prognostic value, these factors may also influence immune competence and systemic inflammatory status. Cancer-related inflammation and hepatic dysfunction have been associated with impaired antitumor immunity and inferior outcomes in hepatobiliary malignancies [1, 2, 11]. ALBI score, in particular, has been shown to reflect both liver function and systemic nutritional–inflammatory status, and has prognostic relevance across hepatobiliary cancers [12, 13]. Similarly, reduced creatinine clearance in the ineligible cohort may reflect not only renal dysfunction per se but also a broader burden of comorbidity and physiological frailty. Renal impairment itself is unlikely to directly reduce exposure to PD-L1 blockade, given the pharmacokinetic properties of monoclonal antibodies [14]. However, advanced chronic kidney disease often reflects a higher burden of comorbidity and frailty; in comparative real-world cohorts, ICI treatment in patients with advanced CKD was not necessarily associated with excess renal toxicity, yet overall survival remained inferior, which may reflect confounding by baseline health status [15].

Consistent with the overall poorer survival observed in the TOPAZ-1-ineligible population, objective response rates were numerically lower in ineligible patients across both treatment groups. In advanced BTC, ORR and DCR have been reported to show only weak correlations with OS [16]. Although response metrics alone may not fully account for survival differences, a generally lower likelihood of tumor shrinkage in the ineligible cohort may reflect more aggressive disease biology and limited treatment sensitivity. In the context of such adverse baseline prognosis, the incremental survival benefit attributable to the addition of durvalumab may have been attenuated or more difficult to detect.

An increased incidence of grade ≥ 3 neutropenia was observed in the GCD group compared with the GC group, regardless of TOPAZ-1 eligibility status. Because durvalumab itself is not generally associated with significant myelosuppression, this difference is unlikely to be directly attributable to the immunotherapy component. Although treatment duration tended to be slightly longer in the GCD group (median, 4.9 vs 4.0 months; *p* = 0.133), the difference was not statistically significant. Instead, differences in clinical practice, including more frequent laboratory monitoring in recent years, may have contributed to increased detection of neutropenia in the GCD group. These findings should be interpreted with caution.

In multivariable analyses stratified by eligibility status, GCD remained independently associated with improved overall survival only among TOPAZ-1-eligible patients, whereas no significant association was observed in the ineligible cohort. Log-transformed CA19-9 consistently emerged as a strong prognostic factor in both strata, highlighting the dominant influence of tumor burden and disease biology on survival. These findings suggest that the magnitude of benefit from immunochemotherapy may depend on host and tumor characteristics aligned with trial eligibility. A similar pattern has been reported in unresectable hepatocellular carcinoma, where adherence to the IMbrave150 inclusion criteria was associated with improved prognosis in real-world patients treated with atezolizumab plus bevacizumab, supporting the broader relevance of trial eligibility in routine practice [17].

From a clinical perspective, the survival benefit of GCD appears to be largely confined to patients meeting the TOPAZ-1 eligibility criteria, whereas its benefit in ineligible patients remains uncertain. In the absence of a clear additional benefit from durvalumab, GC alone may be considered as a treatment option in selected patients; however, these findings do not support a definitive preference for GC over GCD given the retrospective design. Exploratory analyses further suggest that the impact of ineligibility is heterogeneous, with severe anemia and limited life expectancy driving poor outcomes, supporting an individualized treatment approach.

Taken together, our findings support GCD as a standard first-line therapy for advanced BTC, while suggesting that its survival benefit may not be uniform across all real-world patients. Careful patient selection based on functional reserve and baseline clinical characteristics remains essential when translating trial-based evidence into routine practice.

## Limitations

This study has several limitations. First, temporal bias may have influenced the observed outcomes, as GCD was introduced later than GC. In our cohort, patients treated with GCD were predominantly treated in more recent years, whereas those treated with GC were largely treated in earlier periods (Supplementary Table 3). Advances in supportive care, imaging, and treatment strategies over time may have contributed to improved survival outcomes independently of treatment effects. Second, the retrospective design of this study introduces the possibility of residual confounding despite multivariable adjustment. Third, treatment allocation was not randomized and may reflect physician selection bias. Fourth, imaging assessments and adverse event reporting were not centrally reviewed. Fifth, although stratified analyses were prespecified, the study was not powered to formally detect statistical interaction between treatment regimen and eligibility status. Sixth, follow-up duration differed between treatment groups, which may have influenced survival comparisons. Finally, molecular biomarkers, such as PD-L1 expression and tumor mutational burden, were not uniformly available, precluding biological stratification.

## Conclusions

In this large multicenter real-world cohort, GCD was associated with improved overall survival compared with GC in the overall population; however, this association appeared to be primarily observed among patients meeting TOPAZ-1 eligibility criteria, although formal effect modification was not statistically confirmed. These findings suggest that the effectiveness of durvalumab-based immunochemotherapy may vary according to baseline functional and biological characteristics, emphasizing the importance of patient selection when translating clinical trial evidence into routine practice.

## Supplementary Information

Below is the link to the electronic supplementary material.

Supplementary file1 (PPTX 205 KB)

Supplementary file2 (DOCX 23 KB)
