# Supplementary material for: Impact of TOPAZ-1 eligibility on the survival benefit of durvalumab plus gemcitabine–cisplatin in advanced biliary tract cancer: a multicenter real-world study
Source: J Gastroenterol. 2026 Apr 12;61(8):1170–80. doi: 10.1007/s00535-026-02412-6 (PMC13407461; doi:10.1007/s00535-026-02412-6)
Supplement: Supplementary file 1 — Supplementary file1 (PPTX 205 KB) [file 535_2026_2412_MOESM1_ESM.pptx]

## Slide 1
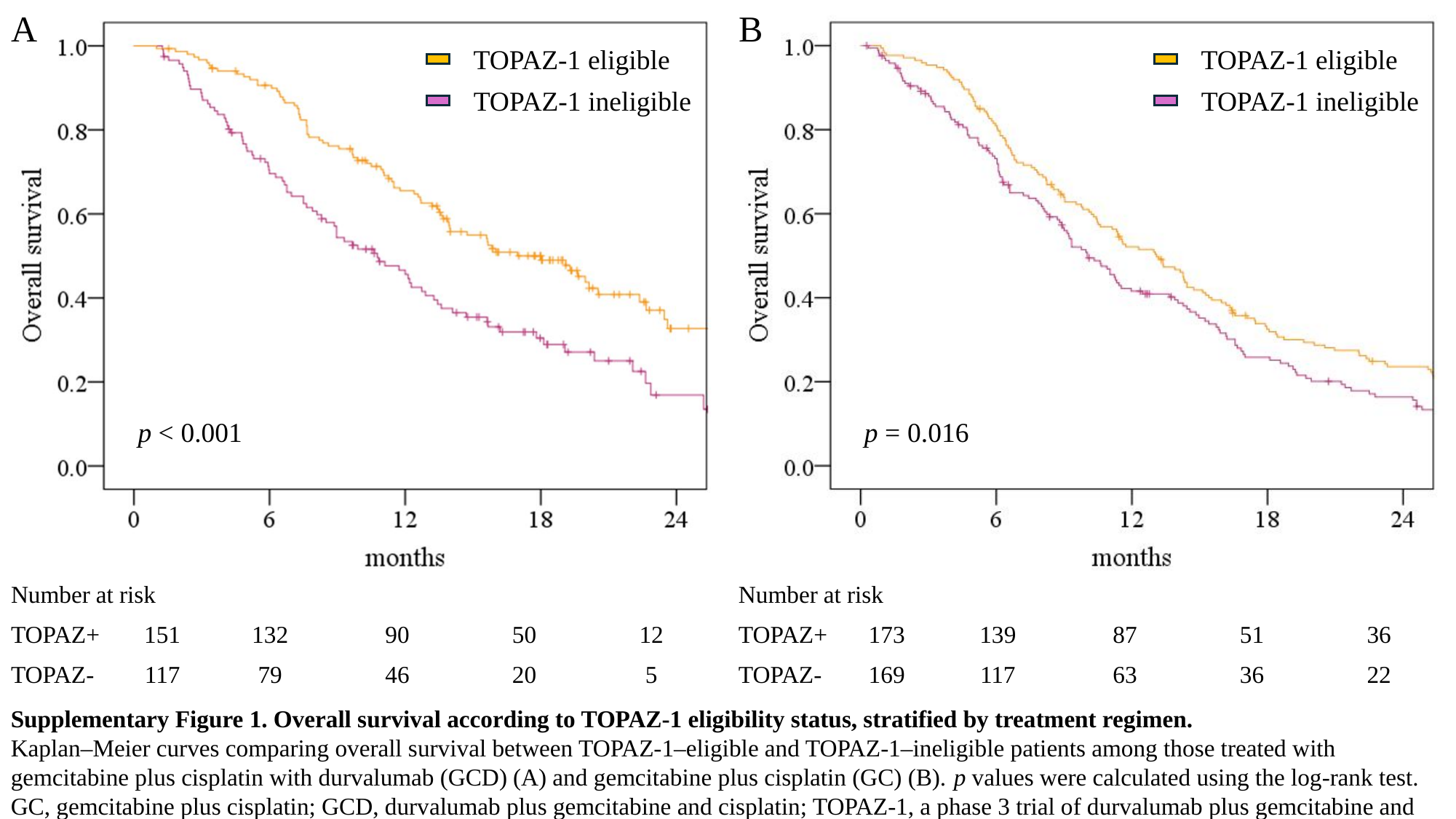

A
B
TOPAZ-1 eligible
TOPAZ-1 eligible
TOPAZ-1 ineligible
TOPAZ-1 ineligible
p < 0.001
p = 0.016
| Number at risk | | | | | |
| --- | --- | --- | --- | --- | --- |
| TOPAZ+ | 151 | 132 | 90 | 50 | 12 |
| TOPAZ- | 117 | 79 | 46 | 20 | 5 |
| Number at risk | | | | | |
| --- | --- | --- | --- | --- | --- |
| TOPAZ+ | 173 | 139 | 87 | 51 | 36 |
| TOPAZ- | 169 | 117 | 63 | 36 | 22 |
Supplementary Figure 1. Overall survival according to TOPAZ-1 eligibility status, stratified by treatment regimen.
Kaplan–Meier curves comparing overall survival between TOPAZ-1–eligible and TOPAZ-1–ineligible patients among those treated with gemcitabine plus cisplatin with durvalumab (GCD) (A) and gemcitabine plus cisplatin (GC) (B). p values were calculated using the log-rank test.
GC, gemcitabine plus cisplatin; GCD, durvalumab plus gemcitabine and cisplatin; TOPAZ-1, a phase 3 trial of durvalumab plus gemcitabine and cisplatin in advanced biliary tract cancer.

## Slide 2
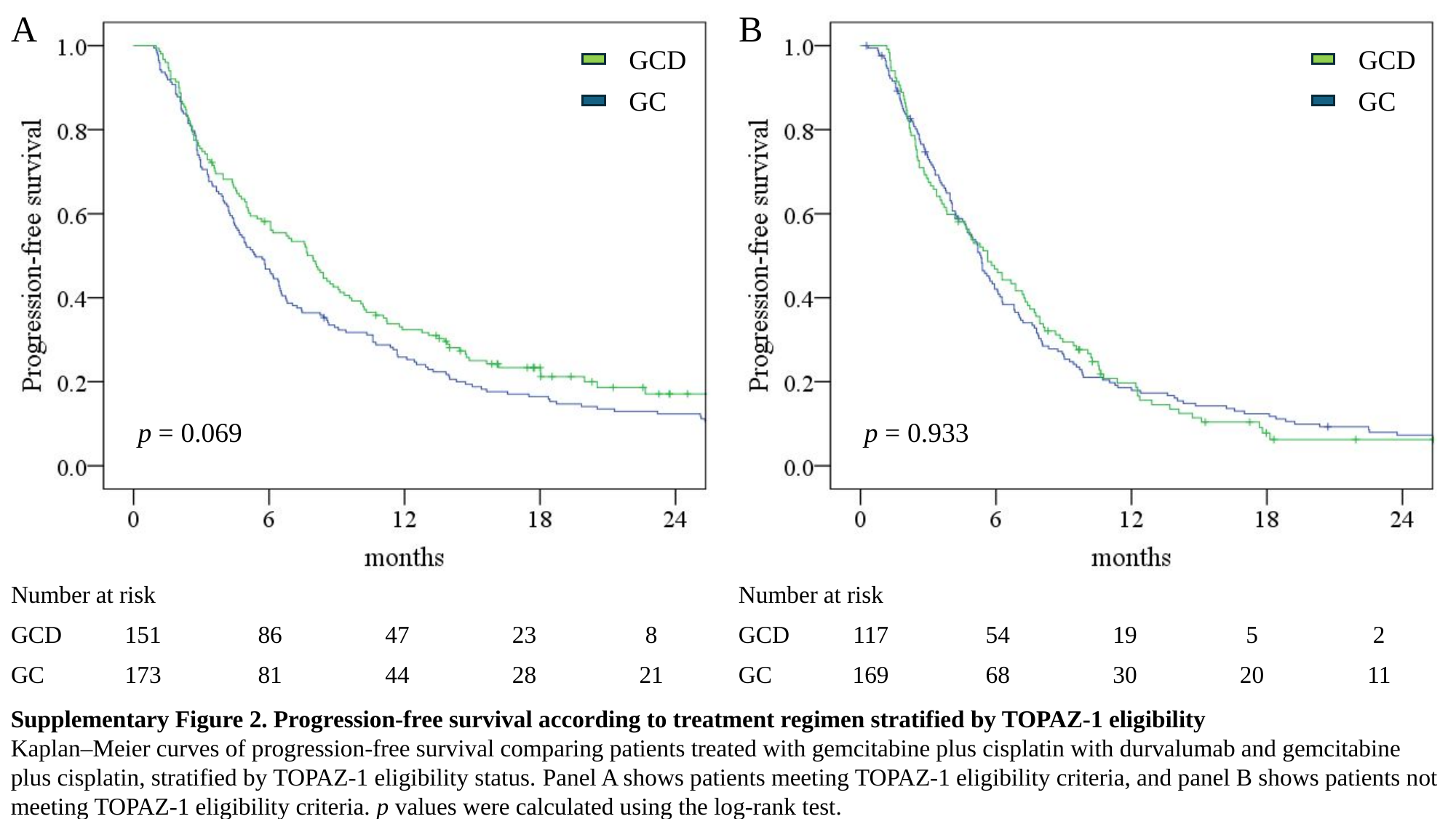

A
B
GCD
GCD
GC
GC
p = 0.069
p = 0.933
| Number at risk | | | | | |
| --- | --- | --- | --- | --- | --- |
| GCD | 151 | 86 | 47 | 23 | 8 |
| GC | 173 | 81 | 44 | 28 | 21 |
| Number at risk | | | | | |
| --- | --- | --- | --- | --- | --- |
| GCD | 117 | 54 | 19 | 5 | 2 |
| GC | 169 | 68 | 30 | 20 | 11 |
Supplementary Figure 2. Progression-free survival according to treatment regimen stratified by TOPAZ-1 eligibility
Kaplan–Meier curves of progression-free survival comparing patients treated with gemcitabine plus cisplatin with durvalumab and gemcitabine plus cisplatin, stratified by TOPAZ-1 eligibility status. Panel A shows patients meeting TOPAZ-1 eligibility criteria, and panel B shows patients not meeting TOPAZ-1 eligibility criteria. p values were calculated using the log-rank test.
GC, gemcitabine plus cisplatin; GCD, durvalumab plus gemcitabine and cisplatin; TOPAZ-1, a phase 3 trial of durvalumab plus gemcitabine and cisplatin in advanced biliary tract cancer.

## Slide 3
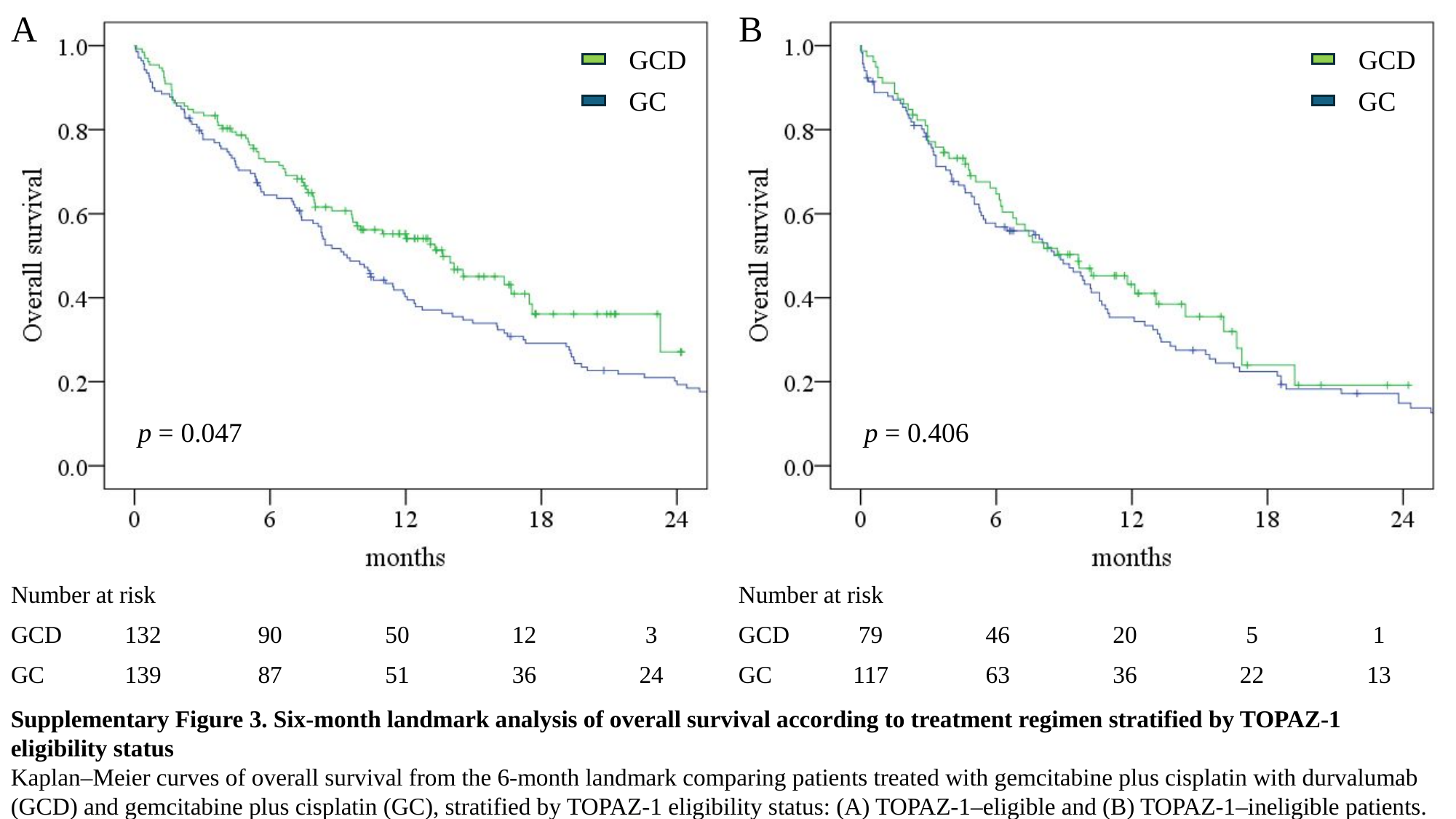

A
B
GCD
GCD
GC
GC
p = 0.047
p = 0.406
| Number at risk | | | | | |
| --- | --- | --- | --- | --- | --- |
| GCD | 132 | 90 | 50 | 12 | 3 |
| GC | 139 | 87 | 51 | 36 | 24 |
| Number at risk | | | | | |
| --- | --- | --- | --- | --- | --- |
| GCD | 79 | 46 | 20 | 5 | 1 |
| GC | 117 | 63 | 36 | 22 | 13 |
Supplementary Figure 3. Six-month landmark analysis of overall survival according to treatment regimen stratified by TOPAZ-1 eligibility status
Kaplan–Meier curves of overall survival from the 6-month landmark comparing patients treated with gemcitabine plus cisplatin with durvalumab (GCD) and gemcitabine plus cisplatin (GC), stratified by TOPAZ-1 eligibility status: (A) TOPAZ-1–eligible and (B) TOPAZ-1–ineligible patients. Only patients alive at 6 months after treatment initiation were included in the analysis. P values were calculated using the log-rank test.
GC, gemcitabine plus cisplatin; GCD, durvalumab plus gemcitabine and cisplatin; TOPAZ-1, a phase 3 trial of durvalumab plus gemcitabine and cisplatin in advanced biliary tract cancer.
