# Supplementary material for: Impact of TOPAZ-1 eligibility on the survival benefit of durvalumab plus gemcitabine–cisplatin in advanced biliary tract cancer: a multicenter real-world study
Source: J Gastroenterol. 2026 Apr 12;61(8):1170–80. doi: 10.1007/s00535-026-02412-6 (PMC13407461; doi:10.1007/s00535-026-02412-6)
Supplement: Supplementary file 2 — Supplementary file2 (DOCX 23 KB) [file 535_2026_2412_MOESM2_ESM.docx]

| **Supplementary Table 1. Reasons for TOPAZ-1 trial ineligibility** | |
| --- | --- |
| Reason for ineligibility | Number (%) |
| Age <18 years | 0 (0.0) |
| ECOG PS ≥2 | 34 (5.6) |
| Non-measurable disease | 68 (11.2) |
| Prior immune checkpoint inhibitor | 0 (0.0) |
| Ampullary carcinoma | 35 (5.7) |
| Hemoglobin <9 g/dL | 29 (4.8) |
| Absolute neutrophil count <1.5 × 10⁹/L | 13 (2.1) |
| Platelet count <100 × 10⁹/L | 19 (3.1) |
| Total bilirubin >2.5 × ULN | 13 (2.1) |
| AST or ALT >2.5 × ULN | 78 (12.8) |
| Creatinine clearance ≤50 mL/min | 91 (14.9) |
| Life expectancy <12 weeks | 4 (0.7) |
| Single eligibility violation | 203 (33.3) |
| Multiple eligibility violations | 83 (13.6) |
| Values are expressed as number (percentage). Percentages were calculated using the total cohort (N = 610) as the denominator. Individual reasons for ineligibility are not mutually exclusive. ECOG PS, Eastern Cooperative Oncology Group performance status; ULN, upper limit of normal; AST, aspartate aminotransferase; ALT, alanine aminotransferase. | |

| **Supplementary Table 2. Exploratory multivariable Cox analysis of individual reasons for TOPAZ-1 ineligibility and overall survival in TOPAZ-1–ineligible patients** | | |
| --- | --- | --- |
| Variable | Hazard ratio (95% CI) | *p* value |
| ECOG performance status ≥2 | 1.460 (0.756–2.822) | 0.260 |
| Non-measurable disease | 1.520 (0.798–2.896) | 0.203 |
| Ampullary carcinoma | 1.223 (0.604–2.477) | 0.576 |
| Hemoglobin <9 g/dL | 2.143 (1.121–4.096) | 0.021 |
| Absolute neutrophil count <1.5 × 10⁹/L | 0.617 (0.250–1.521) | 0.294 |
| Platelet count <100 × 10⁹/L | 0.787 (0.395–1.565) | 0.494 |
| Total bilirubin >2.5 × ULN | 1.657 (0.781–3.516) | 0.188 |
| AST or ALT >2.5 × ULN | 1.603 (0.845–3.041) | 0.149 |
| Creatinine clearance ≤50 mL/min | 0.727 (0.399–1.326) | 0.298 |
| Life expectancy <12 weeks | 6.462 (2.039–20.476) | 0.002 |
| Multiple eligibility violations (≥2 vs 1) | 1.294 (0.975–1.718) | 0.074 |
| Values are presented as hazard ratios with 95% confidence intervals derived from a multivariable Cox proportional hazards model including all variables listed in the table, in patients classified as TOPAZ-1 ineligible (n = 286). All variables were entered simultaneously without selection. ECOG PS, Eastern Cooperative Oncology Group performance status; ULN, upper limit of normal; AST, aspartate aminotransferase; ALT, alanine aminotransferase. | | |

| **Supplementary Table 3. Distribution of treatment periods according to treatment regimen** | | |
| --- | --- | --- |
| Treatment period | GCD (n = 268) | GC (n = 342) |
| ≤2022 | 1 (0.4) | 313 (91.5) |
| ≥2023 | 267 (99.6) | 29 (8.5) |
| Values are presented as number (percentage).  GCD, gemcitabine plus cisplatin plus durvalumab; GC, gemcitabine plus cisplatin. | | |
